# Res-Dom: predicting protein domain boundary from sequence using deep residual network and Bi-LSTM

**DOI:** 10.1093/bioadv/vbac060

**Published:** 2022-09-01

**Authors:** Lei Wang, Haolin Zhong, Zhidong Xue, Yan Wang

**Affiliations:** Institute of Medical Artificial Intelligence, Binzhou Medical University, Yantai, Shandong 264003, China; School of Life Science and Technology, Huazhong University of Science and Technology, Wuhan, Hubei 430074, China; School of Life Science and Technology, Huazhong University of Science and Technology, Wuhan, Hubei 430074, China; Institute of Medical Artificial Intelligence, Binzhou Medical University, Yantai, Shandong 264003, China; School of Software Engineering, Huazhong University of Science and Technology, Wuhan, Hubei 430074, China; Institute of Medical Artificial Intelligence, Binzhou Medical University, Yantai, Shandong 264003, China; School of Life Science and Technology, Huazhong University of Science and Technology, Wuhan, Hubei 430074, China

## Abstract

**Motivation:**

Protein domains are the basic units of proteins that can fold, function and evolve independently. Protein domain boundary partition plays an important role in protein structure prediction, understanding their biological functions, annotating their evolutionary mechanisms and protein design. Although there are many methods that have been developed to predict domain boundaries from protein sequence over the past two decades, there is still much room for improvement.

**Results:**

In this article, a novel domain boundary prediction tool called Res-Dom was developed, which is based on a deep residual network, bidirectional long short-term memory (Bi-LSTM) and transfer learning. We used deep residual neural networks to extract higher-order residue-related information. In addition, we also used a pre-trained protein language model called ESM to extract sequence embedded features, which can summarize sequence context information more abundantly. To improve the global representation of these deep residual networks, a Bi-LSTM network was also designed to consider long-range interactions between residues. Res-Dom was then tested on an independent test set including 342 proteins and generated correct single-domain and multi-domain classifications with a Matthew’s correlation coefficient of 0.668, which was 17.6% higher than the second-best compared method. For domain boundaries, the normalized domain overlapping score of Res-Dom was 0.849, which was 5% higher than the second-best compared method. Furthermore, Res-Dom required significantly less time than most of the recently developed state-of-the-art domain prediction methods.

**Availability and implementation:**

All source code, datasets and model are available at http://isyslab.info/Res-Dom/.

## 1. Introduction

The protein domain is the predominant concept in many areas of the biological sciences. At the sequence level, protein domains are considered to be homologous portions of sequences and are ‘evolutionary units’. In structural terms, domains have generally been observed as local, compact units of protein structure, with a hydrophobic interior and a hydrophilic exterior, and they form a globular-like state that cannot be further subdivided (Keith, 2008). Thus, protein domains are the basic units of protein structure, function, evolution and design, and it is important to divide protein domains accurately.

Due to the importance of the protein domains, many methods have been developed to delineate the domain boundaries. These methods can be divided into two major classes, namely, those that are structure-based and those that are sequence based. Structure-based methods use an experimental structure as input. DomainParser (Guo *et al.*, 2003), PDP (Alexandrov and Shindyalov, 2003), DIAL (Pugalenthi *et al.*, 2005), DDOMAIN (Zhou *et al.*, 2007), DHcL (Koczyk and Berezovsky, 2008) and Sword (Postic *et al.*, 2017) define domains directly using the experimental structures of proteins.

Sequence-based methods can be further divided into three categories: free-modeling-based, template-based and machine-learning-based methods. Free-modeling-based methods are similar to the structure-based method. SnapDRAGON (George and Heringa, 2002), RosettaDOM (Kim *et al.*, 2005) and OPUS-Dom (Wu *et al.*, 2009) predict domains using predicted structure generated by *ab initio* protein folding methods, but these methods are more time-consuming. Template-based methods are conducted to find homologous templates of the query sequence and predict the protein domain of the target sequence based on the query template alignment. For example, SSEP-Domain (Gewehr and Zimmer, 2006) and DomSSEA (Marsden *et al.*, 2002) use the secondary structure elements alignment to find templates and then predict domains. ThreaDom (Xue *et al.*, 2013) takes advantage of multiple threading alignments and generates the profile of domain conservation score that combines information from template domain structures and terminal and internal alignment gaps. For the query sequence which can find homologous templates, the domain assignment from threading alignments achieves significantly higher accuracy than that from ab initio statistical or machine-learning approaches.

The third group of methods is based on machine learning and the representative methods are DOMpro (Cheng *et al.*, 2006), ConDo (Hong *et al.*, 2019), DNN-Dom (Shi *et al.*, 2019) and DeepDom (Jiang *et al.*, 2019). DOMpro (Cheng *et al.*, 2006) trains recursive neural networks with the features of evolutionary information generated by PSI-BLAST (Altschul *et al.*, 1997), predicted secondary structures and predicted relative solvent accessibility. ConDo trains fully connected deep neural networks with the input feature of coevolutionary information extracted from multiple sequence alignment, predicted secondary structure and predicted solvent accessibility. DNN-Dom uses a protein position-specific matrix, 3-state predicted secondary structure, solvent accessibility and amino acid one-hot encoding as input features to train a more complex deep learning model, which consists of multi-scale convolutional neural network layers of different kernel sizes and stacked bidirectional gate recurrent units. The output from these two fully connected layers is further used as deep features for balanced random forest. DeepDom trains a stacked bidirectional long short-term memory (Bi-LSTM) model using a large number of protein sequences without using feature engineering. Recently, Zheng *et al.* (2020) proposed a novel method named FUpred, which can deduce domain boundary from contact map predicted by ResPRE (Li *et al.*, 2019) motivated by the quick progress recently achieved in the field of contact prediction.

The methods based on machine learning have the advantages of fast speed and achieve better performance than template-based methods when there are few homologous sequences that can be found. To further improve the performance of protein domain prediction, based on our originally developed DNN-Dom algorithm, we drew on the ideas of deep residual network and transfer learning in computer vision and natural language processing. The deep residual network was originally used in computer vision and then expanded to the field of computational biology such as protein structure prediction (Wang *et al.*, 2017), cell image recognition (Sadak *et al.*, 2020) and RNA secondary structure prediction (Wang *et al.*, 2021). Transfer learning can recognize and apply knowledge and skills learned in previous domains/tasks to novel domains/tasks and is particularly efficient when data are rich in a source domain but lacking in a target domain. In the natural language processing field, transfer learning often appears in the form of pre-trained language models, such as word2vec (Mikolov *et al.*, 2013). Transfer-learning has succeeded in extracting information from unlabeled sequence databases relevant for various protein prediction tasks (Heinzinger *et al.*, 2019). Recently, Rives *et al.* (2019) developed the protein pre-trained language model called ESM based on Transformer which generates latent embedding acquired from sequence data alone. ESM uses high-dimensional vectors to represent residues in a contextual protein sequence. The output embedding has a multi-scale organization reflecting the structure from the level of biochemical properties of amino acids to the remote homology of proteins. These complex and dynamic feature representations inspired us to improve the prediction of structural domains in proteins.

In this study, an end-to-end deep learning model based on a deep residual neural network and bi-LSTM module, named Res-Dom, was developed to predict the continuous multi-domain protein domain boundary. In addition to the commonly used secondary structure feature, solvent accessibility, and hidden Markov model (HMM) profile generated by HHblits (Remmert *et al.*, 2011), we took advantage of the embedding of the sequences generated by the ESM model. Compared with the current state-of-the-art tools on an independent dataset, Res-Dom achieved superior results in terms of most evaluation metrics. Finally, all source code, datasets and model files can be freely available at http://isyslab.info/Res-Dom/.

## 2. Methods

### 2.1 Datasets

#### 2.1.1 Training and testing datasets

The training dataset was collected from CATH (v4.1) (Dawson *et al.*, 2017). Firstly, all proteins extracted from CATH (v4.1) were filtered out with lengths < 80 or domain lengths < 40. The chains with length >1500 were also removed because SCRATCH (Magnan and Baldi, 2014) which was used to predict the secondary structure and solvent accessibility, was limited by the protein length (<1500). To remove redundant sequences, we used CD-HIT (Huang *et al.*, 2010) with a clustering threshold (sequence identity) of 30%. The final training dataset included 12 306 protein chains. The independent testing datasets were derived from the CATH (4.3), with a sequence identity of >30% to any sequence in the training dataset being removed. After that, there were 342 sequences in the final independent test datasets. The method of dividing datasets by sequence similarity may result in some related proteins in the training set and test set, but at present, this is the most common method. In addition, we used another independent test dataset collected from Critical Assessments of Structure Prediction (CASP)13. After removing protein chains with length < 80 residues or domain length < 40 residues there were a total of 48 target sequences in CASP13 remaining.

#### 2.1.2 Protein sequence features and label assignment

The HMM profile, secondary structure, solvent accessibility and embedding from ESM were chosen as the model input.

The HMM profile was generated by HHblits which searches against uniclust30(2020.06). For a protein sequence of L amino acids, the dimension of the HMM profile was L×30. For each residue, it consisted of 20 emission frequencies, 7 transition probabilities and 3 local diversities.

Secondary structure and solvent accessibility are commonly used features for domain boundary detection (Hong *et al.*, 2019; Jiang *et al.*, 2019; Shi *et al.*, 2019). Solvent accessibility can imply whether a residue is inside or outside the domain. Secondary structure and solvent accessibility were predicted using SCRATCH (Cheng *et al.*, 2005). Secondary structure had three classes (Helix, Strand, Loop) and the dimension of the feature was L×3. The solvent accessibility feature has two classes (Explored, Buried) and the dimension of the feature was L×2.

With the development of pre-trained language models and the emergence of a large number of protein sequences, the study of protein pre-trained models has made great progress recently. Downstream tasks can be improved by using the representation of protein sequences learned from a massive set of unlabeled protein sequences. Hence, in addition to the traditional features, we also employed the embedding from ESM in Res-Dom. The embedding of protein sequences was generated from the pre-trained language model esm1_t34_670M_UR50S which was trained on 250 million protein sequences with up to 670 million parameters. The dimension of the embedding matrix was L×1280. ESM-MSA-1b can extract sequence coevolutionary feature in MSA, and its performance is generally better than ESM-1 (Rao *et al.*, 2021). However, we did not adopt it for two main reasons: first, the computational complexity of ESM-MSA-1b is higher than that of ESM-1, and the complexity of ESM-MSA-1b is $O\left(LM^{2}\right)+O\left(ML^{2}\right)$ and the complexity of ESM-1 is $O\left(L^{2}\right)$, where *M* is the number of rows and L the number of columns in the MSA, and second, ESM-MSA-1b relies on co-evolutionary information in MSA; when the protein does not have enough MSA sequences, protein sequence context embedding of ESM-MSA-1b is inferior to that of ESM-1. To guarantee the generalization performance of our model to these proteins, we adopted ESM-1 as the feature extraction model.

Our network can take variable-length proteins as input. We trained our deep network using a minibatch dataset that is routinely used in deep learning. At each training iteration, we use a minibatch of proteins to calculate gradients and update the model parameters. Considering that most proteins have lengths up to 700 residues, we decided to use a fixed-length window strategy independent of the protein length to encode an input sequence into equal-length fragments (Jiang *et al.*, 2019). The sequence lengths were normalized to 700, with zero padding for less than 700 and truncation for more than 700 residues. After concatenating all the features, the final feature vector was 700×1315.

Because the true domain boundary definitions have not been universally accepted, we assigned all residues within ±20 residues from each true domain boundary residue to the boundary class and the rest to the non-boundary class, according to the conventions used in prior studies (Chen *et al.*, 2010; Marsden *et al.*, 2002). This also alleviated the problem of sample imbalance in the sequence data.

### 2.2 Model architecture

In recent years, with the number of identified protein sequences increasing dramatically, deep learning has become widely used for solving biological problems and has achieved much success, such as protein secondary structure prediction (Spencer *et al.*, 2015; Wang *et al.*, 2016; Yang *et al.*, 2018), protein function prediction (Kulmanov and Hoehndorf, 2020), disorder prediction (Hanson *et al.*, 2017; Wang *et al.*, 2016), contact–contact prediction (Adhikari *et al.*, 2018; Wang *et al.*, 2017), protein–protein or protein–RNA interaction (Hashemifar *et al.*, 2018; Pan and Shen, 2017) and transmembrane prediction (Feng *et al.*, 2020). There are many deep-learning architectures that have been developed to date. The residual neural network (He *et al.*, 2016) is one of the most widely used because it solves the degradation problem of traditional neural networks. The residual network becomes easier to be optimized by adding shortcut connections. The mathematical form of the shortcut connection is as follows:

(1) $$y=H\left(x\right)=F\left(x,W\right)+x,$$

where x and y are the input and output of a residual block, respectively, H(x) represents the residual block, F represents the residual mapping to be learned, and W is the trainable weight parameters. The shortcut connection adds the input and output of the residual block to avoid the problem of vanishing gradients.

**Figure 1.**
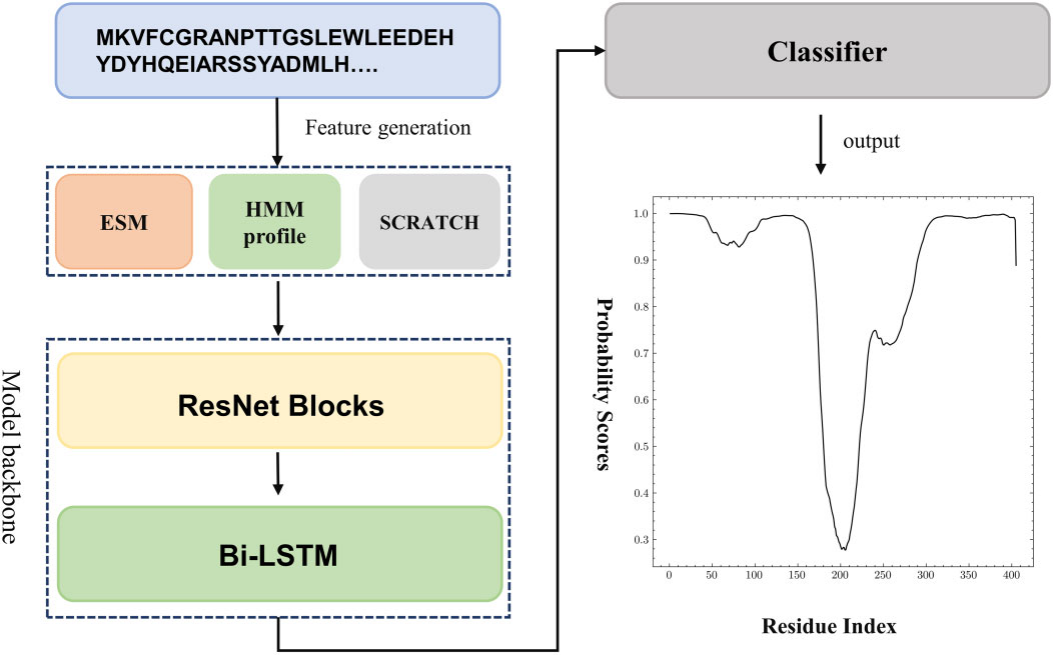
The pipeline of the Res-Dom model

Here, we have developed Res-Dom mainly by improving the model and features based on our DNN-Dom method. There are many intra-domain interactions, which are very helpful for domain boundary prediction. To capture higher-order interactions, we used a deep residual network to extract local and global features. Moreover, to further capture long-range interactions between residues, we added a bidirectional LSTM layer after residual networks. The reason for adopting Bi-LSTM was that it could solve the long-term dependence of RNN by introducing a gating mechanism to control the flow and loss of features, and that bidirectional LSTM could fuse information in different directions of protein sequences. The pipeline of the domain boundary prediction model is illustrated in Figure 1.

**Figure 2.**
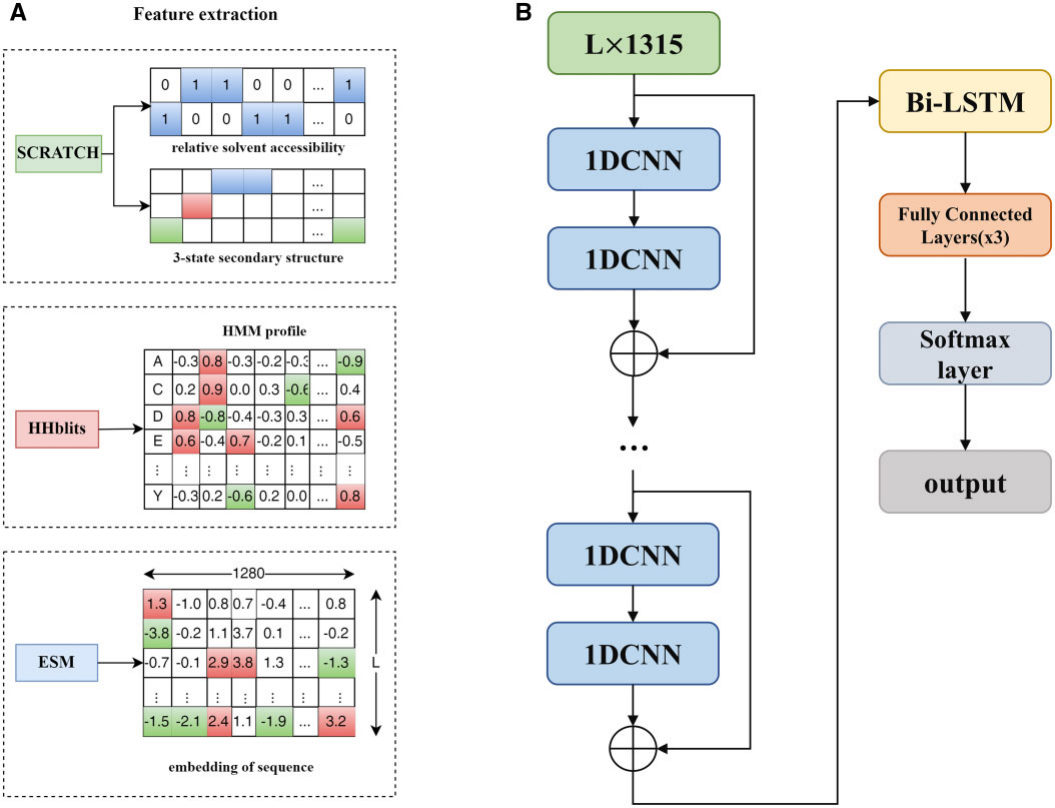
(**A**) Input features included HMM profiles, secondary structures, solvent accessibility and embedding from ESM. (**B**) The model backbone of Res-Dom consisted of 14-layers of ResNet, a Bi-LSTM layer, three fully connected layers and a SoftMax layer

We combined HMM profiles, secondary structures, solvent accessibility and embedding from ESM as input to train this model (Figure 2A). The framework of Res-Dom consisted of a 14-layer ResNet block, the Bi-LSTM, three fully connected layers, and the SoftMax layer (Figure 2B). To balance training time, we chose the 14-layers ResNet architecture. The outputs of ResNet were then fed into the Bi-LSTM to further enhance the long-term dependence of protein sequences. The key to the LSTMs is the cell state that ran straight down the entire chain. The LSTMs have structures called gates that control the information removed or added to the cell state. The ability to avoid vanishing gradients is mainly due to the design of the forget gate in the LSTM. Thus, by training the parameters of the gates, long-range interaction signal between residues may propagate far without significant loss. The outputs of LSTM were fed to three stacked fully connected layers and the SoftMax layer was used to classify the residue feature space, including boundary and non-boundary residues.

Res-Dom takes the entire protein sequence of L residues as input and outputs a vector of L possibility scores, where each score in the vector indicates that the corresponding residue belongs to domain boundary. The loss function is defined as:

(2) $$\mathrm{Loss}\left(y,\hat{y}\right)=-\left(w_{d}y\log\left(\hat{y}\right)+w_{b}\left(1-y\right)\log\left(1-\hat{y}\right)\right)+\lambda \sum_{u}\;u^{2},$$

where *y* and $\hat{y}$ are the label and the predicted possibility scores, respectively; ground truth label y reflects if it belongs to the domain boundary (1) or non-domain boundary (0), and $\hat{y}$ is the output of the model; λ is the regularization factor; u represents all the trainable parameters in the network; $w_{d}$ and $w_{b}$ are the reciprocal of the number of protein residues at domain boundaries and non-domain boundaries, which can alleviate the imbalance of samples in the dataset. In domain boundary prediction, 0.5 was selected as the threshold, residue probability scores greater than 0.5 were considered non-domain boundaries, and those less than 0.5 were considered domain boundary regions. Among them, in the sequence fragments within the range below the threshold, Res-Dom adopted the sequence median as the predicted domain boundary.

### 2.3 Evaluation criteria

To evaluate the performance of Res-Dom, we adopted precision, recall, ACC, and MCC to evaluate the performance of domain number prediction. In addition, normalized domain overlap (NDO)-score (Tai *et al.*, 2005) and domain boundary distance (DBD) score (Tress *et al.*, 2007) were used as criteria to evaluate the performance of protein domain boundary prediction. In this work, precision, recall, Accuracy (ACC), and Matthew’s correlation coefficient (MCC) were calculated as follows:

(3) $$\mathrm{Pre}\left(\mathrm{multi}\right)=\frac{\mathrm{TM}}{\mathrm{TM}+\mathrm{FM}},\mathrm{Rec}\left(\mathrm{multi}\right)=\frac{\mathrm{TM}}{\mathrm{TM}+\mathrm{FS}},$$

(4) $$\mathrm{Pre}\left(\mathrm{single}\right)=\frac{\mathrm{TS}}{\mathrm{TS}+\mathrm{FS}},\mathrm{Rec}\left(\mathrm{single}\right)=\frac{\mathrm{TS}}{\mathrm{TS}+\mathrm{FM}},$$

(5) $$\mathrm{ACC}=\frac{\mathrm{TM}+\mathrm{TS}}{\mathrm{TM}+\mathrm{TS}+M+\mathrm{FS}},$$

(6) $$\mathrm{MCC}=\frac{\mathrm{TM}\times \mathrm{TS}-\mathrm{FM}\times \mathrm{FS}}{\sqrt{\left(\mathrm{TM}+\mathrm{FM}\right)\left(\mathrm{TM}+\mathrm{FS}\right)\left(\mathrm{FM}+\mathrm{TS}\right)\left(\mathrm{TS}+\mathrm{FS}\right)}},$$

where TM/TS represents the number of cases that were correctly predicted to be multi-domain/single-domain proteins, and FM/FS signifies the number of cases that were incorrectly predicted to be multidomain/single-domain proteins. Pre(multi)/Pre(single) represent the precision of multi-domain/single-domain classification and Rec(multi)/Rec(single) symbolize the recall of multi-domain/single-domain classifications.

Moreover, the NDO score (Tai *et al.*, 2005) and the DBD score (Tress *et al.*, 2007), which were used to assess domain splitting in the CASP experiments, were utilized to assess the domain boundary prediction. The NDO score calculates the overlap between the predicted domain regions and true domain regions, while the DBD score was defined as the distance of the predicted domain boundaries from the true domain boundaries, where all linker regions of these domains were considered as the true boundaries.

## 3. Results and discussion

### 3.1 Hyperparameters tuning

Res-Dom was constructed in Python 3.6.5 with Keras 2.2.0, TensorFlow 1.8.0 and ESM 0.1.0 (https://github.com/facebookresearch/esm). For our Res-Dom model, there were some hyperparameters to optimize, including the number of convolution layers, the convolution filter size setting in every residual block, the number of hidden units for LSTM, and the fully connected layer dimensions. Considering the statistics of all sequence lengths and the convenience of subsequent processing and implementation, the sequence lengths were normalized to 700. Sequences longer than 700 were truncated and those shorter than 700 were padded with zeros. The other hyperparameters are optimized according to the performance on the validation set, as follows. The parameters for the ResNet are listed in Table 1. The hidden unit sizes for the bi-LSTMs were 512. The dimensions of the three fully connected layers were 512, 256 and 128, respectively.

**Table 1.** The parameters for the 14-layers of ResNet.

layer name	14-layers
conv1	[3, 643, 64]a×2
conv2	[3, 1283, 128]×2
conv3	[3, 2563, 256]×2
conv4	3, 5123, 512×1

a Convolution filter (3, 64) means that the kernel size was 3 and the filter number was 64.

### 3.2 Ablation study of Res-Dom

To evaluate the contribution of the Bi-LSTM component and ESM, we set up ablative configurations. Five-fold cross-validation was used to evaluate the performance of Res-Dom, Res-Dom w/o ESM and Res-Dom w/o Bi-LSTM on the training dataset. The entire training dataset was randomly split into five subsets containing the same number of chains. The results of the ablative experiment of domain number classification and domain boundary prediction are shown in Table 2.

**Table 2.** Five-fold cross-validation results of Res-Dom, Res-Dom w/o Bi-LSTM and Res-Dom w/o ESM

	Domain number prediction							
Methods	Single-domain		Multi-domain		All		Boundary prediction	
	Pre	Rec	Pre	Rec	ACC	MCC	NDO	DBD
Res-Dom w/o Bi-LSTM	0.824	0.960	**0.897**	0.616	0.841	0.642	0.904	0.860
Res-Dom w/o ESM	0.821	**0.965**	0.892	0.581	0.836	0.623	0.897	0.865
Res-Dom	**0.903**	0.923	0.846	**0.811**	**0.884**	**0.742**	**0.914**	**0.881**

These bold values have been reflected in the 2.3 Evaluation criteria.

It is easy to see that bi-LSTM obviously plays a very critical role in the domain number prediction task. The ACC and MCC scores of Res-Dom w/o Bi-LSTM and Res-Dom increased from 0.841 to 0.884, 0.642 to 0.742, respectively. For the domain boundary prediction, Res-Dom achieved an NDO score of 0.914 and a DBD score of 0.881, which was about 1.1% (0.904) and 2.4% (0.860) higher than Res-Dom w/o Bi-LSTM. These results illustrate that the long-term residue interaction leaned by Bi-LSTM module was also important for the domain boundary prediction. When the input part of ESM was removed, the performance of Res-Dom degraded significantly. The ACC and MCC scores of Res-Dom w/o ESM and Res-Dom increased from 0.836 to 0.884, 0.623 to 0.742, respectively. For the domain boundary prediction, Res-Dom w/o ESM achieved an NDO score of 0.897 and a DBD score of 0.865, which was about 1.9% (0.904) and 1.8% (0.860) lower than Res-Dom. From this ablation experiment, it can be proved that the semantic representation based on the pre-trained model such as ESM can improve the prediction of domain boundaries. To make a fair comparison, we further compared Res-Dom with other state-of-the-art methods on the independent test set.

### 3.3 Comparison with other state-of-the-art methods

Res-Dom was compared with three template-free state-of-art methods (DNN-Dom, ConDo and FUpred) and the template-based method (ThreaDom). It is worth noting that FUpred and ConDo use protein contact maps, while DNN-Dom only uses sequence and sequence profile information as input features to predict domain boundaries. The overall comparison results are shown in Table 3.

**Table 3.** Comparison of the prediction performance of Res-Dom, ThreaDom and three template-free domain boundary predictors on independent test data

	Domain number prediction							
Methods	Single-domain		Multi-domain		All		Boundary prediction	
	Pre	Rec	Pre	Rec	ACC	MCC	NDO	DBD
Res-Dom	0.913	**0.913**	**0.756**	0.756	**0.871**	**0.668**	**0.849**	0.501
DNN-Dom	0.913	0.829	0.619	0.778	0.816	0.568	0.777	0.418
ConDo	0.916	0.817	0.607	0.789	0.810	0.563	0.788	0.487
ThreaDom	0.888	0.881	0.674	0.689	0.830	0.566	0.842	**0.551**
FUpred	**0.937**	0.766	0.566	**0.856**	0.789	0.559	0.807	0.504

These bold values have been reflected in the 2.3 Evaluation criteria.

In terms of domain classification, Res-Dom achieved the highest MCC (0.668), which was about 17.6% higher than DNN-Dom (The rank 1 in three template-free state-of-art domain classification predictors) and 1.80% higher than ThreaDom. Res-Dom also produced the highest ACC (0.871), followed by ThreaDom (0.830), DNN-Dom (0.816), ConDo (0.81) and FUpred (0.789). Considering the individual metrics, Res-Dom achieved the highest recall (0.913) for single-domain proteins and the highest precision (0.756) for multi-domain proteins. Meanwhile, FUpred achieved the highest precision (0.937) for single-domain proteins, the highest recall (0.856) for multi-domain proteins and the lowest MCC (0.559). This implied that FUpred tends to classify targets as multi-domain proteins. The independent test set consisted of 252 single-domain proteins and 90 multi-domain proteins, while 39.8% (136/342) of proteins in the test set were predicted to be multiple-domains by FUpred.

In terms of domain boundary prediction, Res-Dom achieved the highest NDO score (0.849), followed by ThreaDom (0.842), FUpred (0.807), ConDo (0.788) and DNN-Dom (0.777). This indicated that for Res-Dom at least 84.9% of the residues in the predicted domains overlapped with the correct domains, which was 5% higher than that of the best template-free method (FUpred) and was close to ThreaDom. As for DBD score, ThreaDom achieved the highest score (0.551), FUpred and Res-Dom achieved similar DBD scores, which were 0.504 and 0.501, respectively. ConDo and DNN-Dom achieved DBD score of 0.487 and 0.418, respectively. Overall, Res-Dom achieved state-of-the-art performance in terms of most criteria on the independent dataset.

Furthermore, we also tested Res-Dom against the other three recently developed methods ConDo, DNN-Dom and FUpred on the CASP13 datasets. The results are shown in Table 4. In terms of domain number prediction, Res-Dom achieved the highest score in all evaluation metrics. It achieved an MCC of 0.674, which was 36.4% higher than the second-best method ConDo (0.494). Res-Dom also achieved the highest NDO score (0.812), followed by FUpred (0.777), DNN-Dom (0.755) and ConDo (0.722). However, FUpred achieved got the highest DBD score (0.578), followed by Res-Dom (0.532), ConDo (0.504) and DNN-Dom (0.457). In general, Res-Dom also demonstrated the state-of-the-art performance in most criteria on the CASP13 datasets, especially when considering that it took significantly less time than FUpred on the same target.

**Table 4.** Comparison of the prediction performance of Res-Dom and three template-free domain boundary predictors on the CASP13 datasets

	Domain number prediction							
Methods	Single-domain		Multi-domain		All		Boundary prediction	
	Pre	Rec	Pre	Rec	ACC	MCC	NDO	DBD
Res-Dom	**0.963**	**0.788**	**0.667**	**0.933**	**0.833**	**0.674**	**0.812**	0.532
ConDo	0.917	0.667	0.542	0.867	0.729	0.494	0.722	0.504
DNN-Dom	0.839	**0.788**	0.588	0.667	0.750	0.441	0.755	0.457
FUpred	0.95	0.576	0.500	0.933	0.688	0.479	0.777	**0.578**

These bold values have been reflected in the 2.3 Evaluation criteria.

The superior performance of Res-Dom could be mainly attributed to the following two reasons. Firstly, our method distinguished itself from previous methods in that we employed the deep residual neural networks to model sequence relationships and long-term residue interaction leaned by Bi-LSTM module could assist protein boundary prediction. Second, the protein pre-training model can provide more complex high-order semantic features, improve domain prediction performance and reduce the dependence on evolutionary information.

### 3.4 Case study of domain boundary prediction

**Figure 3.**
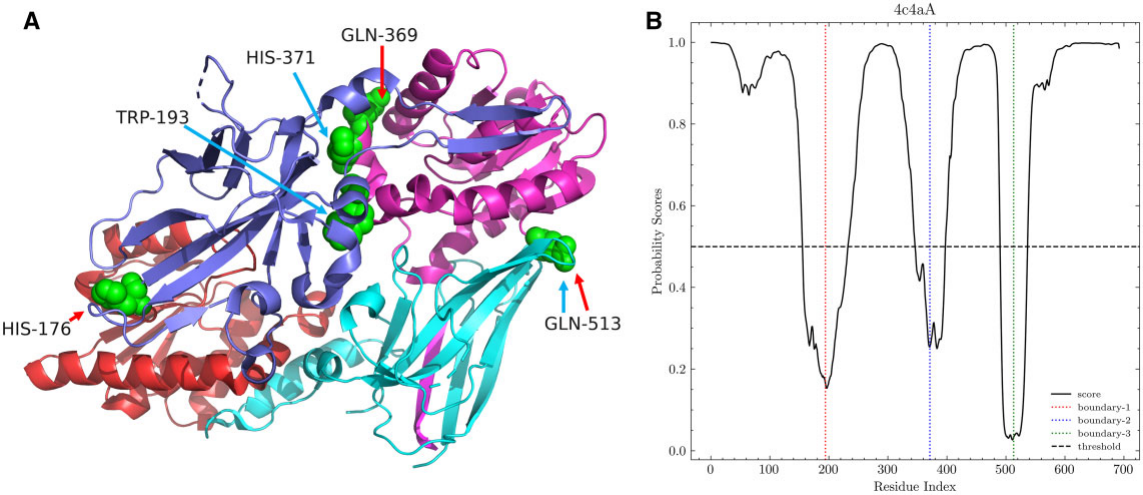
Predicted boundaries for 4c4aA. (**A**) The structure of 4c4aA: the red arrow represents labeled boundaries and blue arrow represents predicted boundaries. (**B**) Predicted probability scores of domain boundaries obtained using Res-Dom

To illustrate the prediction of domain boundaries directly, protein chain 4c4aA of the independent testing datasets was taken as an example of domain boundary prediction and is shown in Figure 3. The protein 4c4aA (PDB code: 4c4a) is an arginine methyltransferase protein, which contains 692 residues and consists of four domains. Protein arginine methyltransferase 7 is a type III arginine methyltransferase, which has been implicated in several biological processes such as transcriptional regulation, DNA damage repair, RNA splicing, cell differentiation and metastasis (Cura *et al.*, 2014). In this case, the real domain boundaries from CATH domain definition were HIS-176, GLN-369, and GLN-513. Judging from the predicted boundary probability score (Figure 3B), there were three boundaries, namely, TRP-193, HIS-371 and GLN-513. Three template-free methods and ThreaDom were also tested on the protein. The result was shown in Table 5. ThreaDom only predicted one correct boundary because ThreaDom cannot find any good template. Although FUpred predicted three domain boundaries correctly, it predicted one more wrong domain boundary. DNN-Dom only predicted one correct boundary and ConDo did not predict any domain boundaries. Within the margin of error (±20), Res-Dom predicted all three boundaries in the 4c4aA protein chain. Although the first predicted boundary was 17 residues away from the real boundary, the remaining domain boundaries were accurately predicted by Res-Dom. Furthermore, the last domain boundary GLN-513 was consistent with the annotation from the CATH database. This example indicates that high-precision domain boundary prediction could be achieved using Res-Dom.

**Table 5.** Comparison between the CATH domain definition of 4c4aA and that predicted by Res-Dom, ThreaDom, FUpred, DNN-Dom and ConDo

Methods	4c4aA
CATH	(1–176)(177**–**369)(370**–**513)(514**–**692)
Res-Dom	(1–193)(194**–**371)(372**–**513)(514**–**692)
ThreaDom	(1**–**374)(375**–**400)(401**–**692)
FUpred	(1**–**36)(37**–**175)(176**–**375)(376**–**511)(512**–**692)
DNN-Dom	(1-225)(226-373)(374-560)(561-692)
ConDo	(1–147)(148–692)

### 3.5 Analysis of time complexity

**Figure 4.**
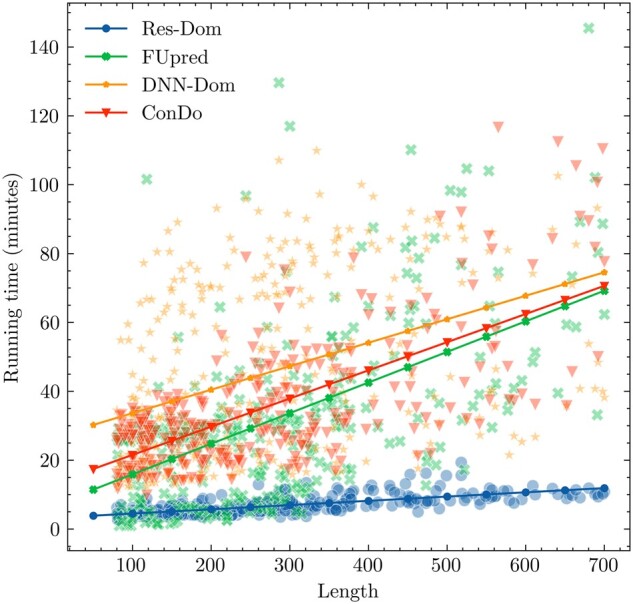
The time complexity comparison between Res-Dom and the other three template-free methods

We compared the time complexity for Res-Dom and the other three template-free methods on the 342 test proteins. All methods run as standalone package with default parameters and the running time for 342 test proteins was calculated on a 20-core CPU. The results are shown in Figure 4. A linear regression function was used to fit the correlation between running time and protein sequence length. DNN-Dom was the most time-consuming method, followed by ConDo, FUpred and Res-Dom. It is worth noting that the running time of Res-Dom does not increase significantly with the increase in sequence length. Overall, Res-Dom had good performance in terms of both accuracy and running time.

## 4. Conclusion

In this work, we developed a novel deep learning model, Res-Dom, based on the deep residual network and took advantage of the embedding generated by ESM. Res-Dom yielded much better performance than other state-of-the-art methods in terms of domain classification. The MCC value of the classified protein domains it achieved was 17.6% and 36.4% than the corresponding second-best method on the independent test dataset and the CASP 13 protein domain dataset, respectively. In terms of domain boundary prediction, Res-Dom also achieved the highest NDO score on both the independent and the CASP13 test datasets, although it got a slightly lower DBD score than FUpred which uses a predicted contact map. It should be noted that FUpred took much more time than Res-Dom on the same target. In sum, Res-Dom obtained the best performance of all the methods compared on the independent test datasets. It also achieved superior results on the CASP 13 protein domain datasets. Discontinuous domains are defined as domains that contain two or more fragments from different regions of the sequence. The prediction of discontinuous domains is still an under-solved problem, ThreaDomEx and FUpred can only partially solve it, mainly due to the fact that they contain some prior knowledge of protein structures, and most tools such as ConDo, DNN-Dom, etc. can only predict the domain boundaries of proteins. The current version of Res-Dom does not consider discontinuous domains, we intend to integrate it with DomEx to generate a more powerful tool which can predict not only domain boundary but also detect discontinuous domains.
